# Progression of renal fibrosis: the underestimated role of endothelial alterations

**DOI:** 10.1186/1755-1536-5-S1-S15

**Published:** 2012-06-06

**Authors:** Dominique Guerrot, Jean-Claude Dussaule, Panagiotis Kavvadas, Jean-Jacques Boffa, Christos E Chadjichristos, Christos Chatziantoniou

**Affiliations:** 1 INSERM U702, Tenon Hospital, Paris, France; 2 Université Pierre et Marie Curie, Paris, France; 3 Service de Néphrologie, CHU Hôpitaux de Rouen, Rouen, France

## Abstract

The vasculature of the kidney is a heterogeneous structure, whose functional integrity is essential for the regulation of renal function. Owing to the importance of the endothelium in vascular biology, chronic endothelial alterations are therefore susceptible to impair multiple aspects of renal physiology and, in turn, to contribute to renal fibrosis. Although systemic endothelial dysfunction is undoubtedly associated with chronic kidney disease, the role of the renal endothelium in the initiation and the progression of renal fibrosis remains largely elusive. In this article, we critically review recent evidence supporting direct and indirect contributions of renal endothelial alterations to fibrosis in the kidney. Specifically, the potential implications of renal endothelial dysfunction and endothelial paucity in parenchymal hypoxia, in the regulation of local inflammation, and in the generation of renal mesenchymal cells are reviewed. We thereafter discuss therapeutic perspectives targeting renal endothelial alterations during the initiation and the progression of renal fibrogenesis.

## Introduction

The kidney receives approximately 20% of the cardiac output, and many essential functions of the organ are supported by the complex organization of renal microvasculature. Therefore primitive or secondary pathological changes in arterioles, glomerular capillaries, vasa rectae and/or peritubular capillaries are susceptible to impair different aspects of renal physiology and, in turn, to contribute to the progression of chronic kidney disease (CKD). Endothelial cells constitute the inner lining of the vessels and are a cornerstone of vascular homeostasis. Besides its classical barrier function, the endothelium is a key player in physiological processes such as the regulation of vasomotor tone, the control of tissue inflammation and of thrombosis [1,2]. Within the renal microvasculature, the endothelium is characterized by a remarkable structural heterogeneity, related to the different and highly specialized functions of endothelial cells, from the preglomerular arterioles to the peritubular capillary bed.

The term "endothelial dysfunction" has been used to define diverse syndromes characterized by changes in distinct endothelial functions, related to a cellular phenotypic switch from a quiescent to an activated state. No clear definition of endothelial dysfunction has been established so far, and this multifaceted disorder actually encompasses a spectrum of disturbances in vasomotor responses, antithrombogenic properties, vascular permeability, leukocyte recruitment and endothelial cell proliferation. In the clinical setting endothelial dysfunction may be detected non-invasively by functional tests evaluating the vasomotor effects of pharmacological substances such as acetylcholine, or of flow-mediated vasodilation after transient ischemia on distal conduit arteries [3,4]. Considerable interest has also been focused on the identification of circulating markers associated with endothelial dysfunction. These include endothelin 1 (ET-1), metabolites of NO (nitrites, nitrates), markers of fibrinolysis and anticoagulant activity (plasminogen activator inhibitor 1, soluble thrombomodulin), and soluble endothelial adhesion molecules (s-E-selectin, s-ICAM, s-VCAM) [5]. More recently circulating endothelial cells, endothelial microparticles and endothelial progenitor cells have been proposed as alternative markers of endothelial cell dysfunction [6].

Cardiovascular outcomes are the major cause of death in end-stage renal disease patients [7]. During the past decade endothelial dysfunction has emerged as an important intermediate factor in CKD. Indeed, with the decreasing glomerular filtration rate, the vasculature is progressively exposed to a burden of pathogenetic conditions responsible for severe functional changes in the endothelium, such as reactive oxygen species (ROS), assymetrical dymethylarginine (ADMA), homocysteine or glycosylated end products [8-11]. We and others have identified ADMA, an endogenous inhibitor of NO synthase (NOS) elevated in CKD patients, as a mediator of endothelial dysfunction, oxidative stress and fibrogenesis [12,13]. Oxidative stress plays an important role in cellular responses to injury, and is a central process in the pathophysiology of endothelial dysfunction. In endothelial cells, ROS can be generated by uncoupled eNOS, which normally produces NO, and lead to the production of oxygen peroxide and subsequent modifications of the cellular phenotype [2,14].

**Figure 1:**
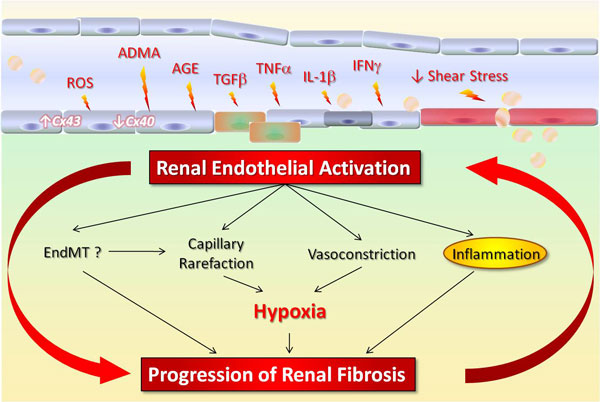
**Schematic view of the pathophysiological role of endothelial activation in chronic kidney disease progression**. (ADMA assymetrical dymethylarginine; ROS reactive oxygen species; AGE advanced glycation end products; TGF transforming growth factor; TNF tumor necrosis factor; IL interleukin; IFN interferon; EndMT endothelial-mesenchymal transition; Cx40 Connexin 40: Cx43 Connexin 43.)

Although the recognition of a systemic endothelial disease related to CKD has led to significant research interest, fewer studies have specifically focused on endothelial alterations within the diseased kidney. We have shown that pharmacological NO deficiency led to ET-1 production in the injured renal endothelial cells with direct profibrotic consequences in the kidney [15]. Recent evidence provides novel insights on the pathophysiological role of intrarenal endothelium in the progression of CKD (Figure 1). In this review we analyze direct and indirect consequences of endothelial alterations on hemodynamics, inflammation and fibrogenesis in the kidney, and discuss therapeutic issues targeting this underestimated culprit in renal fibrosis.

## Review

### Renal endothelial injury contributes to parenchymal hypoxia

Chronic hypoxia mediates the progression of renal fibrosis, even from the early stages of CKD [16]. Interstitial fibroblasts, epithelial cells and endothelial cells develop different responses to hypoxia, which may directly or indirectly contribute to profibrotic mechanisms. Human renal fibroblasts exposed to experimental hypoxic conditions increase collagen production and decrease the expression of extracellular matrix remodeling enzymes [17]. Aerobic oxidative metabolism-dependent epithelial cells physiologically adapt to a reduction in oxygen tension by increasing HIF (hypoxia inducible factor)-dependent signaling, which in turn promotes cell survival [18]. In chronic hypoxic conditions these tubular adaptive mechanisms may be overrun and cell function and viability compromised.

Although the endothelium receives direct oxygen supply from red blood cells, global or regional hemodynamic disturbances may also be responsible for endothelial hypoxia and activate the endothelial cells. In a rat model of renal artery clamping with rescue by non-injured endothelial cells, Brodsky and colleagues identified acute endothelial dysfunction as a contributor to the hemodynamic "no-reflow" phenomenon in post-ischemic kidneys [19]. In this setting, endothelial activation and subsequent adhesion of leukocytes to the endothelium was believed to create hemodynamic resistances which reduce regional blood flow.

Peritubular capillaries constitute the major network supplying oxygen to the nephrons. Therefore capillary functional alterations, by reducing blood flow or leading to subsequent reduction of vascular density, are susceptible to be a major determinant of kidney disease progression. Consistently, studies on human biopsies have associated microvascular rarefaction with the progression of renal failure, while evidence for a pathogenetic role of capillary endothelial alterations in CKD progression *via *chronic tubulointerstitial hypoxia stems from experimental models. In renal biopsies of CKD patients, Choi and colleagues have demonstrated that morphological alterations of peritubular capillaries were strongly associated with parameters of tubulointerstitial injury [20]. Similarly a histomorphological evaluation of cadaveric kidney allografts performed at the time of transplantation showed that sustained kidney injury is associated with a reduction in Von Willebrand Factor staining on peritubular capillaries (but not on renal arteries or arterioles) [21]. In experimental renovascular disease therapeutic prevention of vascular rarefaction by intrarenal administration of VEGF leads to the preservation of renal blood flow and a reduction in subsequent fibrosis [22]. Together with several other studies, these results suggest straightforward pathophysiological consequences of peritubular endothelial alterations on both short- and long-term aspects of renal function.

Interlobular arteries and afferent arterioles are the major site for renal blood flow autoregulation. Since endothelial cells control vasomotor tone in these resistance vessels, in particular *via *NO and endothelium-derived hyperpolarizing factors, alterations of the endothelium may also contribute to glomerular hemodynamic disturbances and to downstream hypoxia in the kidney. Fawn-Hooded hypertensive (FHH) rats present defective renal blood flow autoregulation and develop progressive kidney failure with age. Ochodnicky and colleagues have studied endothelium-dependent vasodilation in this inbred model of spontaneous renal disease and identified that endothelial dysfunction in the renal artery precedes the development of kidney damage [23]. Although the mechanistic explanations for these results presumably differ from those related to chronic peritubular endothelium injury, this study establishes that the integrity of preglomerular endothelium is required for renal vascular homeostasis.

Overall, current evidence underlines renal endothelial hypoxia as the actor of an important profibrotic vicious circle in CKD progression (Figure 1).

### Endothelial functional alterations promote tissue inflammation

Exaggerated inflammation has long been identified as a major player in acute and chronic renal diseases, including allograft rejection, glomerulonephritis, ischemia-reperfusion or cisplatin-induced nephropathy. The endothelium is at the interface between blood and circulating cells on the one hand, and tissue on the other hand. In physiological and pathological conditions, endothelial cells integrate mechanical stimuli related to local hemodynamics and cytokinic stimuli such as IFN-gamma, IL-1beta or TNF-alpha. The latter cytokines are able to induce the expression of distinct patterns of adhesion molecules on the luminal surface, thereby promoting a site- and cell-specific recruitment of circulating leukocytes. Schematically leukocyte recruitment is a three-step process. Leukocytes first roll on the activated endothelium, via the interaction between selectins (E-selectin, P-selectin) and leukocyte surface antigens. Firm adhesion is then facilitated by the endothelial expression of ICAM-1 and VCAM-1, which also promote subsequent transmigration of the leukocytes into the tissue, together with PECAM/CD31.

In the kidney pathophysiological implications of endothelial selectins and adhesion molecules have been demonstrated in a large variety of clinical settings and experimental models. In an ischemic model of renal failure, P-selectin blockade with monoclonal antibodies improved renal function and the administration of fucoidan, an oligosaccharide which non-specifically inhibits P-selectin, increased renal blood flow [24]. Similarly, transgenic mice lacking basigin/CD147, a ligand for E-selectin, exhibited reduced kidney damage after ischemia-reperfusion as compared to control mice, indicating a critical role for E-selectin in neutrophil recruitment in this setting [25]. In rodents, the injection of allo-immune nephrotoxic serum induced the expression of endothelial selectins and leukocyte rolling in glomeruli and capillaries, an event that was significantly blunted by fucoidan in postcapillary venules [26,27].

By using anti-ICAM-1 monoclonal antibodies in rats and ICAM-1 transgenic mice, Kelly and colleagues have shown that this adhesion molecule plays an early and important pathogenetic role in renal ischemia-reperfusion, *via *neutrophil infiltration, with both functional and structural consequences on the kidney [28,29]. Adhesion of circulating cells to the endothelium of renal vessels may also contribute to potentially severe hemodynamic alterations, which can further aggravate tissue injury. Overall these studies underline the importance of endothelial activation in the genesis of local inflammation at early stages of inflammatory kidney diseases.

Gap-junctional intercellular communication is essential in the coordination and integration of microvascular function by the endothelial cells in a very complex manner. Gap junctions are composed of intercellular channels formed by connexins (Cx), which allow the direct exchange of ions, small metabolites and other second messenger molecules between adjacent cells [30]. Each type of Cx-made channel has a unique inherent gating property, or permeability to various molecules. Therefore, Cx composition of gap junction channels appears to determine selectivity among second messengers [31].

Alterations of the expression of endothelial Cx have been associated to the development of chronic and acute vascular inflammatory diseases [32]. For example, a decrease in the expression of Cx40 has been reported in the endothelial layer covering atherosclerotic lesions in the aorta of hypercholesterolemic mice. Mice in which Cx40 was specifically deleted from the endothelium showed enhanced monocyte infiltration at the very early stages of the disease [33]. In contrast to Cx40, Cx43 expression seems to be upregulated in the dysfunctional endothelium and endothelium-specific deletion of Cx43 in mice greatly reduced the TNF-α-induced leukocyte adhesion and transmigration [34]. It has also been recently suggested that this Cx may serve as a conducting pathway by amplifying Ca^2+^-signalling between endothelial cells to spread inflammatory signals within the lung capillary network [35]. In addition, Cx43^+/- ^mice were protected against chronic or acute inflammatory disease, as they displayed reduced inflammatory cell recruitment at the injured site [32]. Interestingly, in accordance with the above mentioned studies, we recently observed a marked increase of the expression of Cx43 in the endothelium of peritubular and glomerular capillaries at the early stages of hypertension-induced renal disease in mice. The Cx43 expression pattern was paralleled closely by that of adhesion markers such as VCAM-1 and ICAM-1, known to play a major role in the recruitment of inflammatory cells [36]. Although further work is required to clarify the implication of Cx in renal inflammation, the Cx hypothesis may be of interest in the recruitment of inflammatory cells in the kidney, as several studies showed an important role of these proteins in the inflammatory response.

### Endothelial-mesenchymal transition: a new player in renal fibrosis?

The origin of renal fibroblasts is an evolving and somewhat controversial issue [37-39]. Acquisition of a mesenchymal-like phenotype by tubular epithelial cells, named epithelial-mesenchymal transition (EMT), has been largely studied in renal fibrosis over the past decade. More recently several research teams have suggested that endothelial cells may also acquire functional and structural characteristics of mesenchymal cells after tissue injury. This so-called endothelial-mesenchymal transition (EndMT) had previously been recognized in normal conditions, particularly during embryonic development of the heart. Increasing evidence now argues that EndMT may play an additional role in a variety of diseases, including in kidney injury [40].

*In vitro*, endothelial cells of different origins can acquire a mesenchymal phenotype, spontaneously or after stimulation by TGF beta. In a model of cardiac overload in *Tie2-Cre;ROSA-STOP-lacZ *transgenic mice, the presence of markers usually expressed by myofibroblasts (FSP-1, alpha SMA) was partially co-localized with the endothelial fate tracer lacZ, suggesting the endothelial origin of heart myofibroblasts [41]. Interestingly mice treated with human recombinant BMP-7 appeared to be protected against both EndMT and the development of cardiac fibrosis.

During the past 4 years, an EndMT-like process has also been evidenced in the kidney in several independent experimental models (unilateral ureteral obstruction, pharmacological inhibition of eNOS, streptozotocin-induced nephropathy, *Col4a3*-deficient mice) [42-44]. We have also evaluated the modifications of endothelial-cell phenotype during the progression of angiotensin II-induced nephropathy in Sprague Dawley rats, and observed a limited and focal co-expression of FSP1 with the endothelial marker RECA1, which suggests the presence of EndMT in peritubular renal capillaries in hypertensive nephropathy. Interestingly, features consistent with EndMT were observed at early stages of the renal disease, before the onset of significant proteinuria or fibrosis (Guerrot *et al*., unpublished data). Recently, Basile and collegues have analyzed the phenotypic alterations of endothelial cells after ischemia-reperfusion in *Tie2-Cre;YFP *transgenic mice [45]. The results of this study suggested that the reduced density of endothelial cells following renal ischemia may be due, in part, to EndMT.

Together, these data identify EndMT as a novel aspect of endothelial dysfunction and show that EndMT may be associated with kidney disease. Furthermore, they suggest that endothelial cells which acquire mesenchymal characteristics may directly contribute to early pathogenetic mechanisms of fibrogenesis. However, owing to the heterogeneity of renal endothelial cells, further studies would be of major interest to better understand the relative importance, the molecular mechanisms and local consequences of EndMT, especially with respect to the different compartments of renal microvasculature.

### Targeting renal endothelial dysfunction as an innovative therapeutical approach in renal fibrosis

Since current evidence underlines the importance of endothelial alterations in CKD progression, the prevention of renal endothelial injury emerges as a promising treatment strategy in kidney diseases. Depending on the clinical setting a reduction of endothelial dysfunction is susceptible to alleviate inflammation, hemodynamic disturbances, hypoxia and extracellular matrix synthesis. In renal experimental models, interesting studies have suggested beneficial effects of therapies based on selectin inhibitors, ET-1 antagonists or treatments increasing NO bioavailability [15,46-48]. These strategies all target distinct aspects of endothelial pathophysiology, thereby reducing specific consequences of renal endothelial functional alterations. In a different approach several teams have also shown promising results when promoting replacement of the injured endothelium, either by increasing resident endothelial cell proliferation, stimulating endogenous progenitor mobilization or directly injecting autologous endothelial progenitor cells into the kidney [49-51].

An important characteristic of endothelial activation in kidney diseases is *de novo *expression of surface antigens. Importantly, critical modifications of the endothelial phenotype predominantly occur in the injured region. Therefore, specifically targeting the activated endothelial cells may allow selective delivering of drugs to the diseased vascular bed. This strategy has proven to be efficient in preliminary studies using liposomes conjugated to anti-E-selectin antibodies, to address dexamethasone in a model of allo-immune nephrotoxic serum-induced renal disease [27,48].

## Conclusion

Endothelial dysfunction is a multifaceted disorder that plays a central role in complications related to kidney diseases. Beside the well established systemic endothelial dysfunction associated with CKD, recent evidence highlights direct implications of renal endothelial activation in fibrogenesis. Together these data suggest that the altered renal endothelium may be a promising therapeutic target and prompt further studies to evaluate novel specific strategies for the treatment of CKD progression. Specifically, the possibility of targeted delivery based on endothelial alterations opens novel therapeutic avenues for the treatment of renal diseases. Future research directions may also include inhibition of EndMT, preservation of endothelium-derived hyperpolarizing factors, pharmacological maintenance of eNOS activation in altered shear stress conditions, and silencing of endothelial proinflammatory genes.

## Competing interests

The authors declare no conflict of interest regarding the issues addressed in this review.

## Authors' contributions

DG drafted the manuscript. JCD, JJB, CEC and CC helped to draft the manuscript. DG and CC revised the manuscript. All authors read and approved the final manuscript.
